# Supplementary material for: The causal relationship between the human gut microbiota and pyogenic arthritis: a Mendelian randomization study
Source: Front Cell Infect Microbiol. 2024 Nov 26;14:1452480. doi: 10.3389/fcimb.2024.1452480 (PMC11629706; doi:10.3389/fcimb.2024.1452480)
Supplement: Supplementary file 1 [file DataSheet1.zip › mendelian test/ebi-a-GCST90027636.csv_forest.pdf]

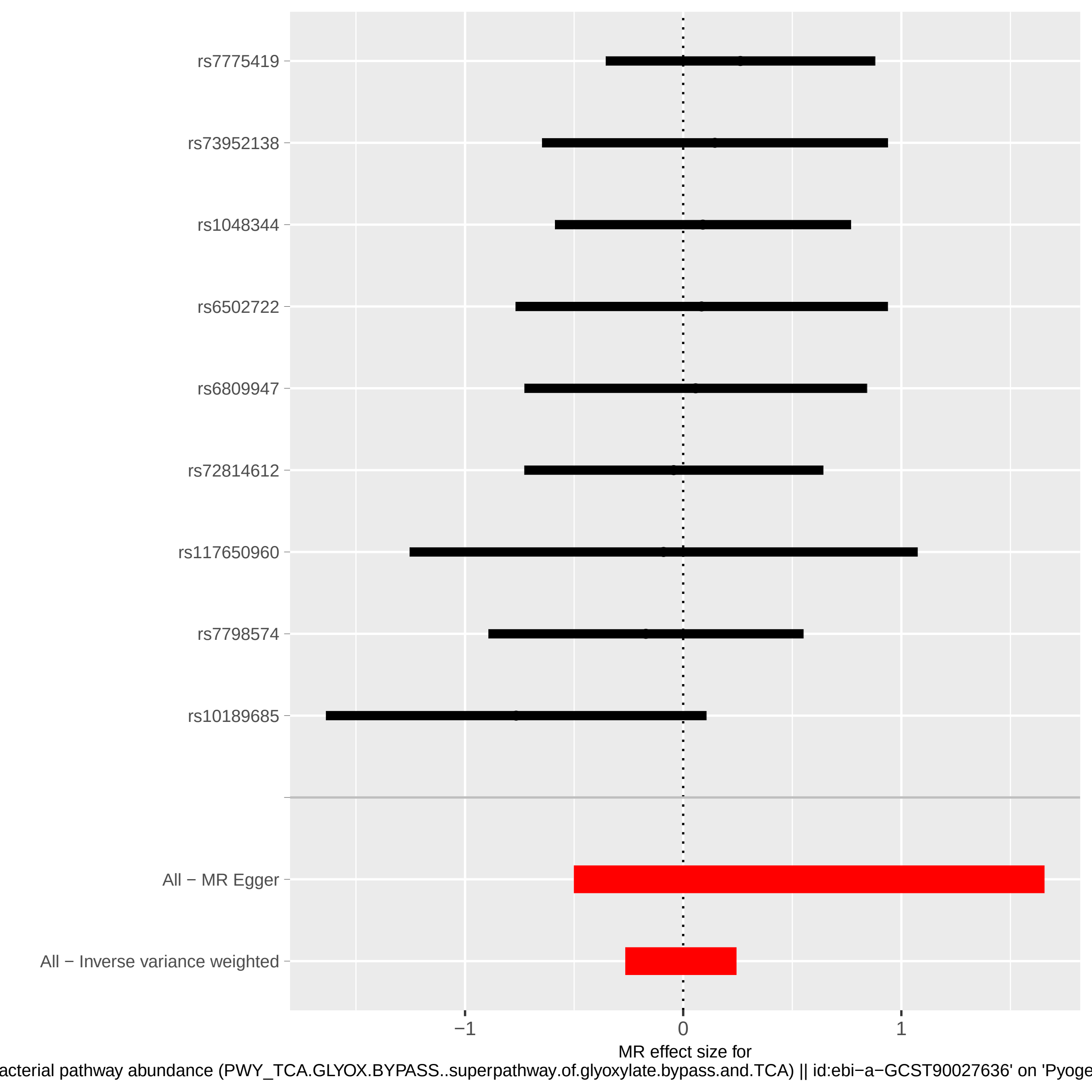
rs7775419
rs73952138
rs1048344
rs6502722
rs6809947
rs72814612
rs117650960
rs7798574
rs10189685
All − MR Egger
All − Inverse variance weighted
−1
0
1
MR effect size for
acterial pathway abundance (PWY_TCA.GLYOX.BYPASS..superpathway.of.glyoxylate.bypass.and.TCA) || id:ebi−a−GCST90027636' on 'Pyoge
